# Early recognition of sepsis-associated acute kidney injury based on BioBERT-BPNN multimodal clinical data analysis

**DOI:** 10.1371/journal.pone.0357602

**Published:** 2026-09-11

**Authors:** Yufeng Chen, Hongyang Bian, Yuye Li

**Affiliations:** Shandong Public Health Clinical Center, Shandong University, Shandong, China; University of Diyala College of Medicine, IRAQ

## Abstract

**Background:**

Sepsis-associated acute kidney injury (SA-AKI) is a common and serious complication in intensive care units, marked by high incidence, rapid disease progression, and poor clinical outcomes. Due to its complex pathophysiology and nonspecific clinical manifestations, early recognition of SA-AKI remains highly challenging.

**Methods:**

This study aims to achieve early recognition of SA-AKI using clinical records of sepsis patients. A dual-center dataset of sepsis patients is constructed by integrating 1,000 cases from the publicly available MIMIC-IV database and 400 cases from the electronic medical records system of Shandong Public Health Clinical Center. Multimodal data are generated for each sepsis case by extracting 33 risk factors from multi-source clinical information. A BioBERT-BPNN multimodal model is developed, in which the BioBERT module extracts semantic features from textual data, the BPNN module captures nonlinear features from numerical data, and MLP module is applied for multimodal feature fusion and classification.

**Results:**

The proposed model demonstrates stable and reliable performance for early recognition of SA-AKI on the MIMIC dataset and exhibits good cross-center generalizability and robustness on the external Hospital dataset. Dual-center data enhances the model’s adaptability to local cases and improves its clinical applicability. Moreover, multimodal data have complementary value, the multimodal model outperforms single-modality models.

**Conclusion:**

The proposed BioBERT-BPNN multimodal model effectively integrates textual and numerical information and improves the performance of early SA-AKI recognition. Leveraging dual-center multi-source heterogeneous clinical data enhances its applicability in real-world clinical settings. This study could provide valuable reference for timely intervention, optimized decision-making, and improved outcomes in sepsis patients at risk of AKI, holding important clinical research value.

## 1. Introduction

Acute kidney injury (AKI) is a global severe condition that poses a serious threat to human health and life, defined as a rapid decline in renal excretory function [1]. The global epidemiological study in 2018 showed that the incidence rate of AKI among inpatients was about 20%, while the incidence rate in intensive care units (ICU) was higher, more than 50% [2]. Sepsis, defined as a dysregulated host response to an infection that leads to life-threatening organ dysfunction, is a common serious condition in ICU with high morbidity and mortality [3].

Sepsis is a major risk factor for AKI [4]. Sepsis-associated acute kidney injury (SA-AKI) is defined as AKI occurring within 7 days after the onset of sepsis or septic shock, accounting for approximately 40%−70% of all AKI cases in ICU [5]. Compared with patients with sepsis or AKI alone, those with SA-AKI have significantly worse prognoses and substantially higher healthcare expenditures [5,6].

The complex pathophysiology and nonspecific clinical manifestations of SA-AKI make its early recognition particularly challenging [7]. Traditional diagnosis of SA-AKI primarily depends on delayed indicators such as serum creatinine and urine output, which usually exhibit significant changes only when kidney injury has already been severe [8]. In recent years, the development of electronic medical records (EMR) and the accumulation of clinical data have enabled computer technologies – particularly machine learning and deep learning – to demonstrate great potential in medical data mining and disease classification.

Therefore, this study aims to develop a multimodal data analysis model for the early recognition of SA-AKI by integrating multi-source clinical data from dual-center sepsis patients. By identifying high-risk sepsis cases before AKI onset, this research is expected to enable precise clinical interventions and individualized treatment strategies. The contributions of this work are as follows:

(1) Sepsis case information from MIMIC-IV database and Shandong Public Health Clinical Center EMR are integrated to increase sample diversity, enhancing the generalizability and clinical applicability for SA-AKI cases in our hospital.
(2) Structured multimodal clinical data combining textual data and numerical variables are generated by 33 risk factors from multi-source clinical information to enrich case descriptions and capture potential risk signals.
(3) A BioBERT-BPNN multimodal model is developed to analyze SA-AKI clinical data by combining semantic deep mining with nonlinear feature modeling, enhancing feature representation and improving the performance and robustness.
(4) This work identifies high-risk patients through analysis of clinical descriptions and subtle numerical fluctuations, contributing to the early clinical intervention and reducing both the incidence and severity of SA-AKI.

## 2. Related works

In recent years, research on the early recognition of SA-AKI has increasingly focused on the development of machine learning–based early recognition and prediction models, yielding significant advances [9,10]. Commonly used machine learning algorithms include Logistic Regression [11–13], XGBoost [14, 15], and Random Forests [16]. Some studies have developed more powerful ensemble models by integrating multiple machine learning methods [17,18]. Most studies of SA-AKI early recognition are based on case data from the publicly available MIMIC-III/IV ICU databases [19,20], while a smaller proportion utilize hospital EHR datasets. In these SA-AKI early recognition and prediction models, the most frequently used predictors are serum creatinine, lactate, age, blood urea nitrogen and a history of diabetes, while some studies have incorporated as many as 16 variables for risk scoring [11].

Deep learning methods have also demonstrated great potential in research on SA-AKI. He et al. developed a prognostic prediction model for SA-AKI by RNN-LSTM architecture, which performed better than machine learning models based on decision trees and logistic regression [21]. Mao et al. developed a domain-specific language model for AKI trained on AKI clinical notes, demonstrating superior performance in early AKI recognition compared with general text classification models [22].

## 3. Materials and methods

### 3.1. Data source

To enhance the reliability and generalizability of the research, dual-center sepsis case data were analyzed in this study. Clinical data of sepsis patients were obtained from the publicly available MIMIC-IV database [20] and the Shandong Public Health Clinical Center EMR system. The MIMIC-IV database contains extensive ICU data from multiple hospitals in the United States, characterized by a large sample size and comprehensive clinical variables, and is considered representative of the typical features of critical illness internationally. Shandong Public Health Clinical Center is a comprehensive tertiary Class A hospital located in Shandong Province, China. Sepsis cases from Shandong Public Health Clinical Center exhibit distinct regional characteristics, and their clinical data reflect real-world clinical practice better.

The diagnostic criteria for sepsis were based on the Sepsis-3.0 consensus [3], and the diagnosis criteria for AKI followed the KDIGO guidelines [1]. Sepsis patients with age ≥ 18 and ≤90 years old were eligible for inclusion. Exclusion criteria were as follows: (1) patients who voluntarily discontinued treatment; (2) patients with missing or incomplete key clinical data.

Based on the above criteria, 1000 sepsis patients with complete clinical data were randomly selected from the MIMIC-IV database, including 500 SA-AKI cases and 500 sepsis-unrelated acute kidney injury (non-SA-AKI) cases. Similarly, 400 sepsis patients, including 200 SA-AKI cases and 200 non–SA-AKI cases, were selected from discharged sepsis patients in the Shandong Public Health Clinical Center EMR system from 01/01/2020–31/12/2025.

All clinical data were derived from de-identified records collected during routine clinical diagnosis and treatment. No additional interventions were performed, and no identifiable personal information was disclosed. The use of hospital EMR data was approved by the Ethics Committee of Shandong Public Health Clinical Center (approval number: GWLCZXEC-SOP-K-2025–63). The requirement for informed consent was waived by the ethics committee because this was a retrospective study using de-identified clinical data.

### 3.2. Data extraction

Many risk factors have been implicated in the occurrence of AKI among sepsis patients in ICU, but consensus across studies remains lacking. Through a literature review, 33 main risk factors related to SA-AKI were identified, which were classified into 6 categories: demographic characteristics, disease severity scores, comorbidities, acute complication, treatment interventions and laboratory examinations [23–26]. Details of the specific risk factors and their classification are presented in Table 1.

**Table 1:** Risk factors related to SA-AKI.

Category	Risk Factors
Demographic Characteristics	Sex, Age, BMI
Disease Severity Scores	SOFA, SAPS II, GCS
Comorbidities	Coronary heart disease, COPD, Hypertension, Diabetes, Cerebral infarction, Chronic renal failure, Liver disease, Trauma, ARDS
Treatment Interventions	Invasive mechanical ventilation, Vasopressors
Laboratory Examinations	Red blood cells, Hemoglobin, White blood cells, Platelets, ALT, Bilirubin, Albumin, Creatinine, BUN, Blood glucose, Prothrombin time, pH, Potassium, Sodium, Calcium, Lactate

BMI: body mass index; SOFA: Sequential Organ Failure Assessment; SAPS II: Simplified Acute Physiology Score II; GCS: Glasgow Coma Scale; COPD: chronic obstructive pulmonary disease; ARDS: acute respiratory distress syndrome; ALT: alanine aminotransferase; BUN: blood urea nitrogen.

Information on these 33 risk factors was extracted from the clinical records of sepsis patients, distributed across multiple data sources including admission notes, progress notes, physician orders, discharge summaries and laboratory reports. The integration of multi-source clinical information allows mutual complementation, enriches case representations, and facilitates the capture of complex pathophysiological interactions and potential risk signals.

Baseline characteristics of the selected sepsis cases from the MIMIC-IV database and Shandong Public Health Clinical Center are presented in S1 Table.

### 3.3. Generation of structured data

**Fig 1:**
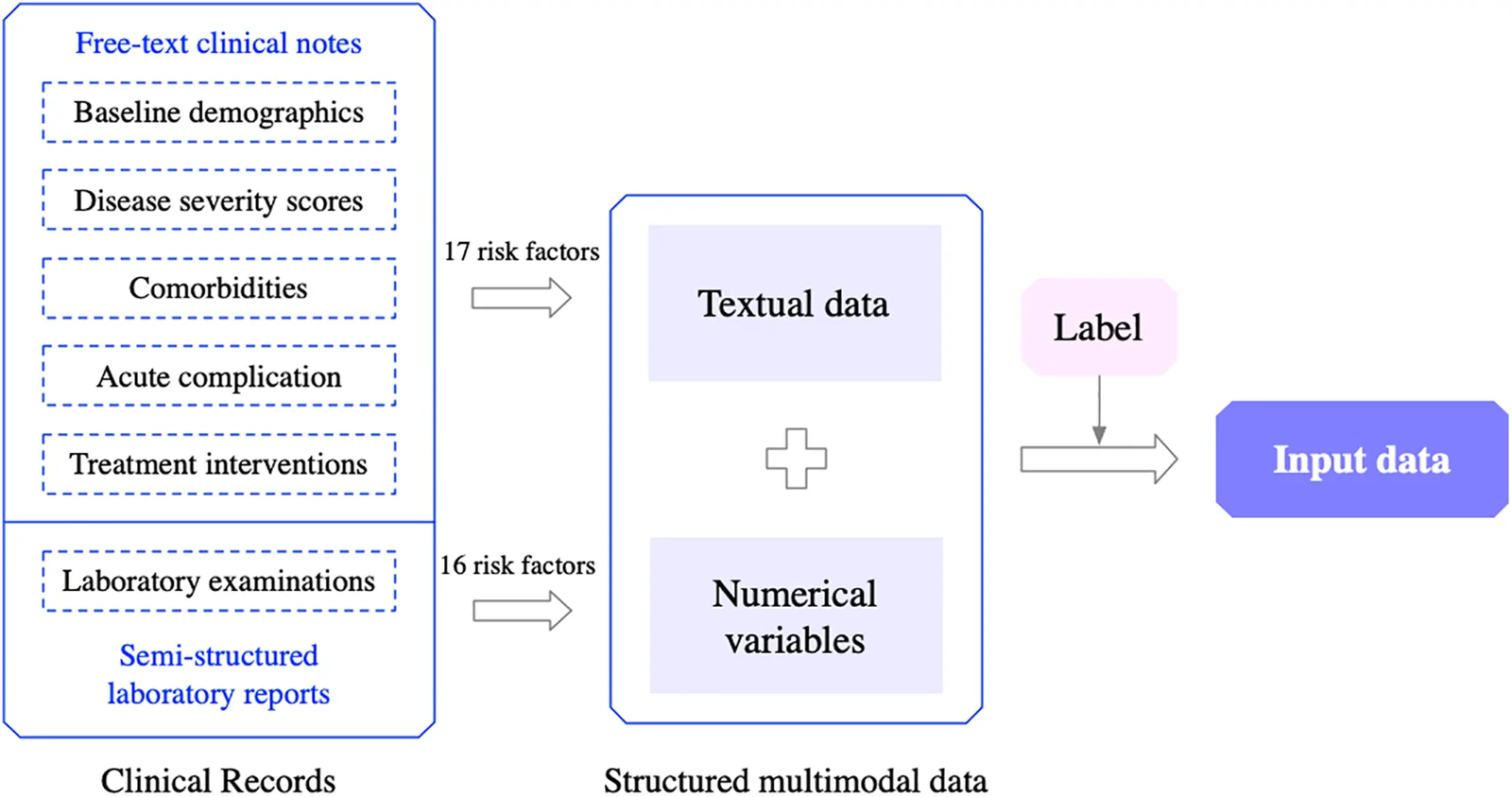
Flowchart of the structured multimodal data generation process.

This study aimed to characterize high-dimensional patient states from complex clinical records of sepsis cases, identify hidden patterns to explore associations between sepsis and AKI, thereby enabling the early recognition of SA-AKI. Clinical records of sepsis patients mainly consist of free-form clinical text notes and semi-structured laboratory reports. Therefore, a multimodal data structure was developed, consisting of two complementary modalities: textual data and numerical variables, as illustrated in Fig 1.

The textual modality comprised 17 risk factors covering demographic characteristics, disease severity scores, comorbidities, acute complications, and treatment interventions. Textual data were standardized a fixed descriptive format as follows, where the options or placeholders in square brackets were completed using patient-specific information corresponding to the 17 predefined risk factors.

- This sepsis case involves a [male/female] patient, aged [XXX], with a BMI of [XXX], a SAPS score of [XXX], a SOFA score of [XXX], and a GCS score of [XXX]. [He/ She] [has/ has no] coronary heart disease, [has/ has no] chronic obstructive pulmonary disease (COPD), [has/ has no] hypertension, [has/ has no] diabetes, [has/ has no] acute respiratory distress syndrome (ARDS), [has/ has no] chronic kidney disease (CKD), [has/ has no] liver dysfunction, [has/ has no] cerebral infarction and [has/ has no] trauma. The patient [received invasive mechanical ventilation and vasopressors/ did not require invasive mechanical ventilation but received vasopressors/ received invasive mechanical ventilation but did not require vasopressors/ did not require invasive mechanical ventilation or vasopressors].

The numerical modality comprised 16 laboratory indicators spanning eight dimensions: infection, liver function, kidney function, hematology, coagulation, acid-base balance, electrolytes, and tissue perfusion. These variables provided a comprehensive profile of the physiological status of sepsis patients and robust support for subsequent model development.

Finally, outcome labels were generated for chosen sepsis cases. SA-AKI patients were labeled as 1, while non–SA-AKI patients were labeled as 0.

### 3.4. BioBERT-BPNN multimodal model

**Fig 2:**
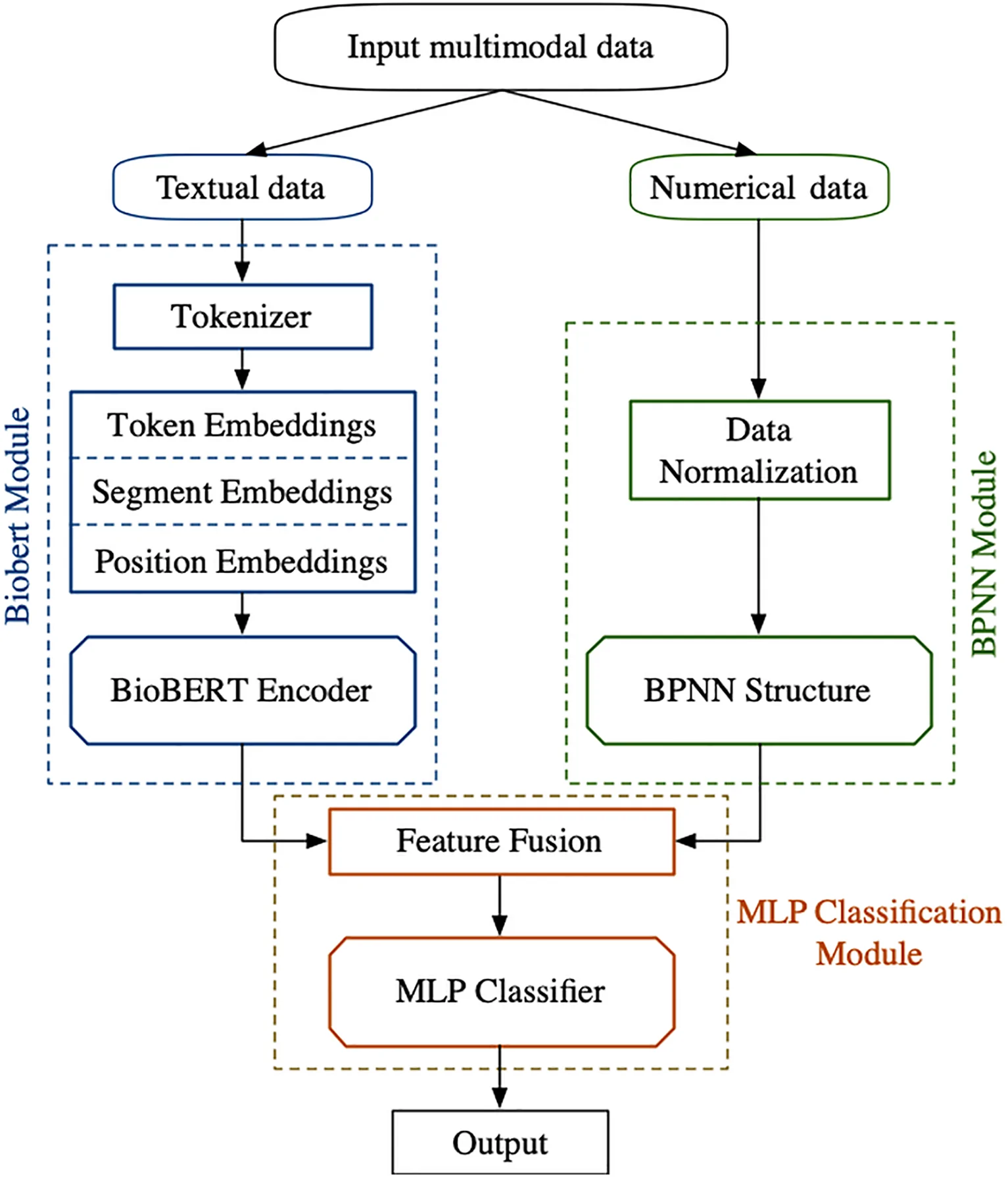
Overview of the BioBERT-BPNN multimodal model.

This work proposes a BioBERT-BPNN multimodal model that jointly exploits textual and numerical clinical information for the early recognition of SA-AKI in sepsis patients, as illustrated in Fig 2. Within this model, textual data are encoded by the domain-pretrained Bidirectional Encoder Representations from Transformers for Biomedical Text Mining (BioBERT) module to generate compact semantic representations, while numerical data are modeled through the Back Propagation Neural Network (BPNN) module to extract nonlinear features. The outputs of both branches are integrated in the multilayer perceptron (MLP) module for final classification.

**Fig 3:**
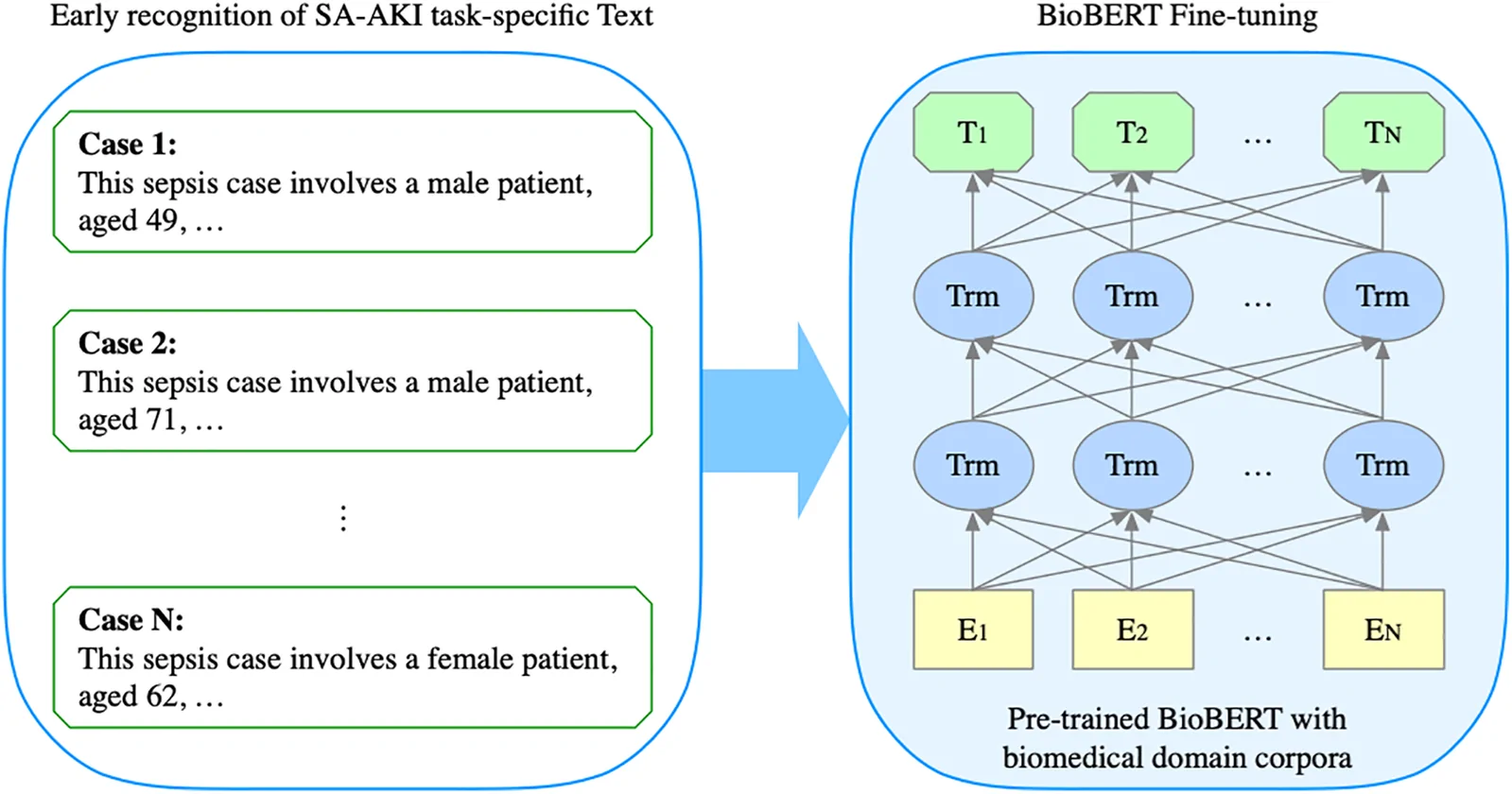
Domain-adaptive fine-tuning of BioBERT.

BioBERT, a domain-specific extension of BERT [27], is further pre-trained on large-scale biomedical text corpora such as PubMed abstracts and PMC full-text articles [28]. By incorporating medical semantic priors, BioBERT retains the strong representational power of the Transformer [29] while significantly improving its ability to address domain-specific challenges in the biomedical field, and has become the strong baseline for biomedical natural language processing (NLP) tasks. Therefore, BioBERT is employed as the encoder for the textual branch of the proposed multimodal model. During the model training with structured clinical text data of sepsis patients, BioBERT is domain-adaptive fine-tuned to improve its applicability for the early recognition of SA-AKI, as shown in Fig 3. This process enables the model to better capture the semantics of sepsis-related narratives and enhances both the efficiency and accuracy in processing clinical records.

**Fig 4:**
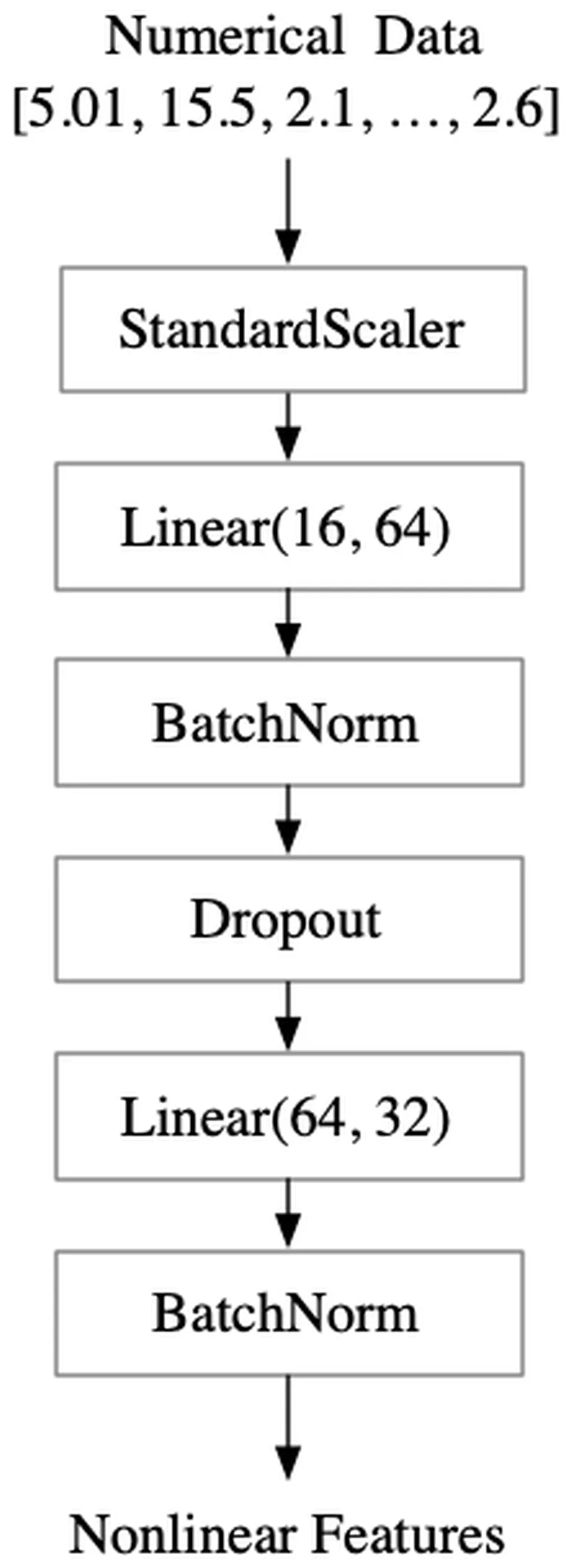
Architecture of the BPNN in the numerical branch.

BPNN is a representative feedforward neural network that iteratively optimizes model performance through forward propagation and weight adjustment via backpropagation. In the numerical branch of the proposed multimodal model, a BPNN with two hidden layers was adopted to extract nonlinear representations from laboratory indicators, as depicted in Fig 4.

The BioBERT module outputs 768-dimensional textual semantic features, which are much higher in dimensionality compared with the 32-dimensional nonlinear numerical features extracted by the BPNN module. To address the dimensional disparity between two modalities, a linear projection is applied to reduce the dimensionality of textual semantic features from 768 to 128, thus harmonizing the feature scales and balancing their contributions in the fusion stage. Multimodal features are generated by concatenating the dimension-reduced textual semantic features with the numerical nonlinear features, representing high-dimensional patient states in sepsis cases. Multimodal features are fed into a two-layer MLP classifier to obtain the final classification results.

## 4. Experiments

### 4.1. Experimental setup

During the experiments, dual-center sepsis cases are utilized, comprising the MIMIC dataset (500 SA-AKI and 500 non–SA-AKI cases) and the Hospital dataset (200 SA-AKI and 200 non–SA-AKI cases). To ensure the reproducibility and comparability, random seed is applied for dataset partitioning, allocating 80% of the data to training and 20% to testing. The random seed for dataset partitioning is set to 42.

During model training, the AdamW optimizer is employed. The initial learning rate is set to 2e-5 for the BioBERT module and 1e-3 for the BPNN and MLP classifier modules. The remaining hyperparameters are configured as follows: β is set to (0.9, 0.999), eps is set to 1e-8, and weight decay is set to 0.01. Training is performed with a batch size of 32 for 100 epochs, and early stopping is applied to prevent overfitting. For the BioBERT module, the maximum sequence length is set to 128. To reduce computational cost and alleviate overfitting, the parameters of the first 5 Transformer encoder layers (out of 12) in BioBERT are frozen, enabling the deeper layers and task-specific heads to concentrate on learning downstream features.

### 4.2. Evaluation metrics

For model performance evaluation, Accuracy, F1-score, and Area Under the Curve (AUC) are selected as the primary metrics. Accuracy measures the overall correctness of prediction, the calculation process is defined as follows:


$$\begin{array}{c} \text{Accur}\text{acy}=\frac{\text{TP}+\text{TN}}{\text{TP}+\text{TN}+\text{FP}+\text{FN}} \end{array}$$ (1)

where TP (True Positive) refers to correctly predicted positive cases; TN (True Negative) refers to correctly predicted negative cases; FP (False Positive) refers to incorrectly predicted positive cases; FN (False Negative) refers to incorrectly predicted negative cases.

F1-score provides a more reliable assessment under class imbalance by the harmonic mean of precision and recall, the calculation process is defined as follows:


$$\begin{array}{c} \text{F1}=\frac{\text{2}\times \text{Precision}\times \text{Recall}}{\text{Precision}+\text{Recall}} \end{array}$$ (2)

$$\begin{array}{c} \text{Precision}=\frac{\text{TP}}{\text{TP}+\text{FP}} \end{array}$$ (3)

$$\begin{array}{c} \text{Recall}=\frac{\text{TP}}{\text{TP}+\text{FN}} \end{array}$$ (4)

where precision refers to the proportion of true positive cases among all cases predicted as positive, reflecting the model’s ability to avoid false positives; recall (sensitivity) refers to the proportion of true positive cases correctly identified among all actual positive cases, reflecting the model’s ability to capture true positives.

AUC refers to the area under the ROC (Receiver Operating Characteristic) curve and evaluates the overall discriminative ability of the model across thresholds. The ROC curve is a plot of True Positive Rate (TPR) against False Positive Rate (FPR) at different classification thresholds. As a supplement, the ROC curve is plotted to provide a comprehensive assessment of the model’s classification performance. The ROC curve and AUC are defined as:


$$\begin{array}{c} \text{TPR}=\text{R}\text{ecall}=\frac{\text{TP}}{\text{TP}+\text{FN}} \end{array}$$ (5)

$$\begin{array}{c} \text{FPR}=\frac{\text{FP}}{\text{FP}+\text{TN}} \end{array}$$ (6)

$$\begin{array}{c} \text{ROC}=\left\{\left(\text{FPR}(t),\text{TPR}(t)\right)|t\in [\text{0},\text{1}]\right\} \end{array}$$ (7)

$$\begin{array}{c} AUC=\int_{\text{0}}^{\text{1}}\text{TPR}\left(\text{FPR}\right)d\left(\text{FPR}\right) \end{array}$$ (8)

where t is the decision threshold.

### 4.3. Experimental results and analysis

The experimental results of the proposed BioBERT-BPNN multimodal model for early recognition of SA-AKI are summarized in three parts. During training, the checkpoint with the highest AUC on the test set is selected and retained for subsequent evaluation.

#### (1) Single-center validation.

**Fig 5:**
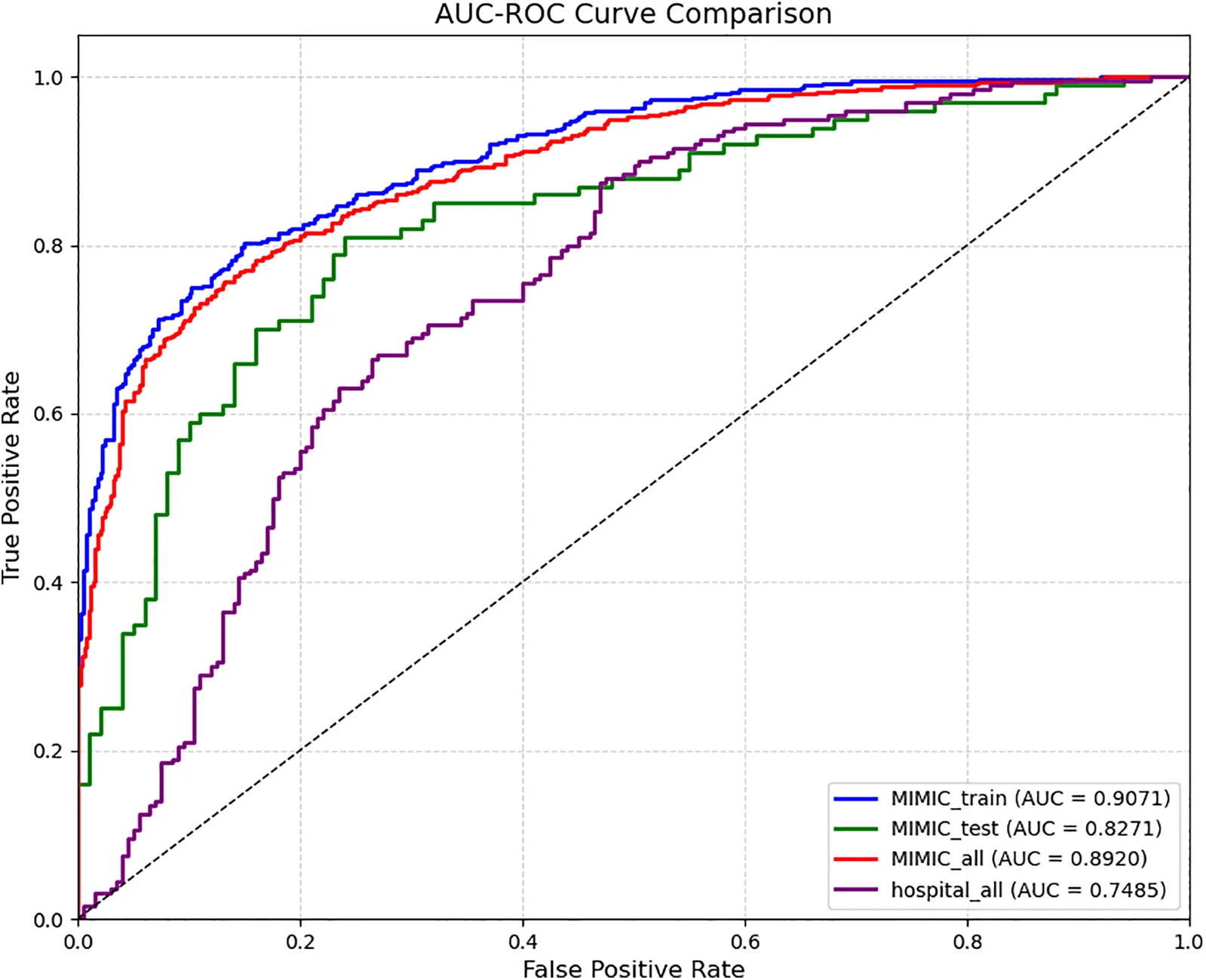
AUC-ROC curve comparison in single-center validation experiments.

The first part is single-center validation. Split the MIMIC dataset into training set and test set, the BioBERT-BPNN multimodal model is trained on the MIMIC-training set and evaluates its performance in early SA-AKI recognition and generalizability on the MIMIC-test set, MIMIC dataset and Hospital dataset. The experimental results are presented in Table 2 and Fig 5.

**Table 2:** Experimental results of single-center validation.

Dataset	Accuracy	F1-score	AUC
MIMIC-training set	0.8225	0.8086	0.9071
MIMIC-test set	0.7550	0.7461	0.8271
MIMIC	0.8110	0.7983	0.8920
Hospital	0.6925	0.6886	0.7485

As shown in Table, the proposed BioBERT-BPNN multimodal model demonstrates satisfactory performance on the MIMIC dataset, with Accuracy, F1-score, and AUC all showing stable and reliable classification capability for early SA-AKI recognition. When externally validates on the Hospital dataset, the model nearly maintains consistent performance. Despite variations in case distribution and local characteristics, it exhibits strong cross-center generalizability and robustness. Fig 5 shows the overall performance of the BioBERT-BPNN multimodal model across different datasets, providing an intuitive depiction of its generalization ability, robustness, and cross-domain adaptability.

#### (2) Multi-center validation.

In the second experimental part, multi-center validation is performed by merging the MIMIC and Hospital datasets into an entire dataset. The proposed model is trained and evaluated on this combined dataset to assess the impact of multicenter data on SA-AKI recognition.

**Fig 6:**
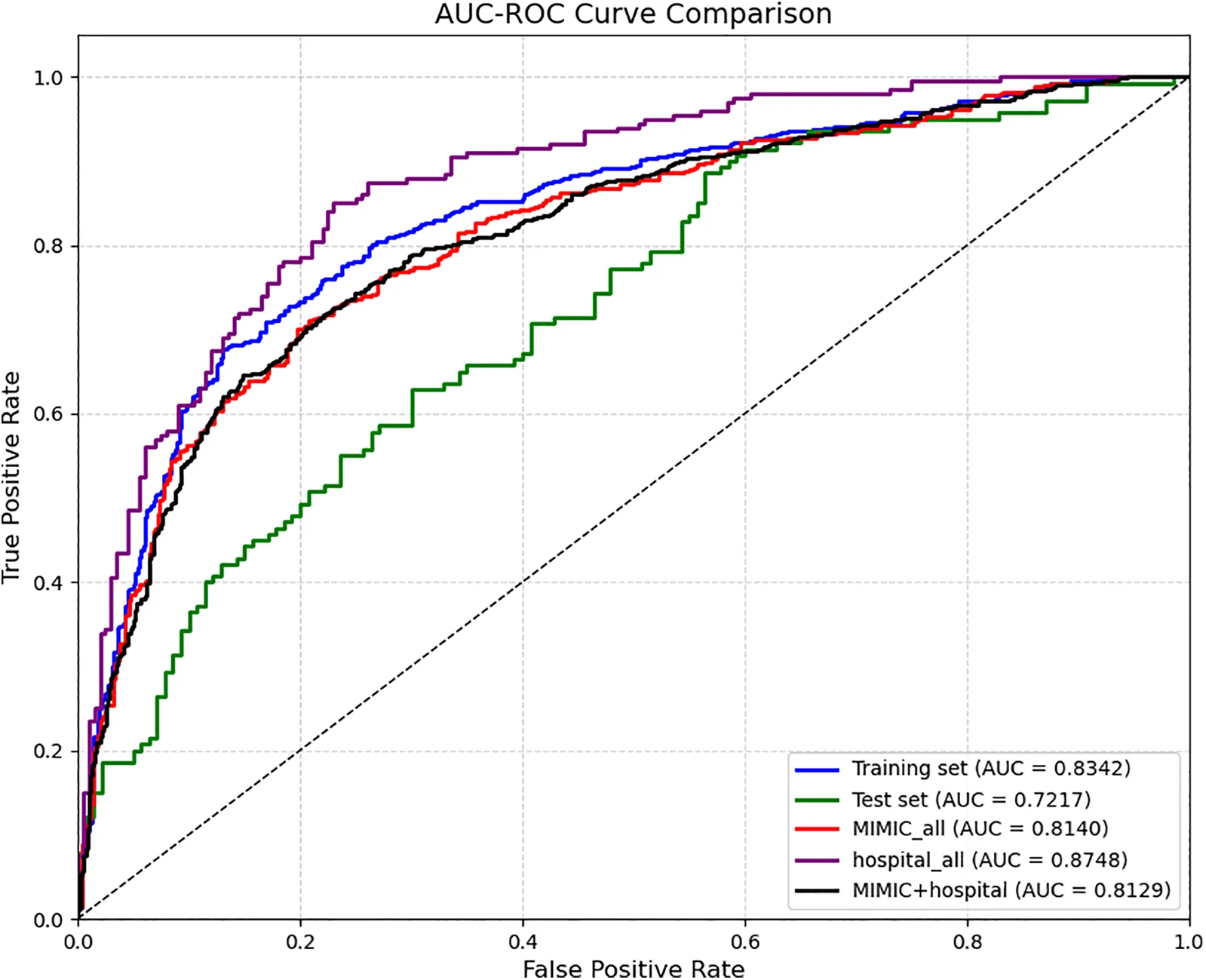
AUC-ROC curve comparison in multi-center validation experiments.

Table 3 and Fig 6 present the early recognition performance of the proposed BioBERT-BPNN multimodal model for SA-AKI on the dual-center datasets from MIMIC and the hospital. Comparison of multicenter validation with single-center validation reveals that incorporating hospital data into the training set slightly reduces model performance in identifying SA-AKI among sepsis cases from the MIMIC database, but significantly improves performance in local hospital cases. This phenomenon can be explained by population differences, variations in disease spectra, and inconsistencies in data distribution between datasets. Specifically, the MIMIC data mainly represents Western ICU populations, whose clinical characteristics and treatment practices differ from those in domestic hospitals. In contrast, incorporating local hospital data improves the model’s adaptability and generalizability to the local patient population. Therefore, although the model performance on multi-center datasets is modestly compromised, the model’s practical value in the target application scenario (i.e., the local hospital setting) is substantially enhanced.

**Table 3:** Experimental results of multi-center validation.

Dataset	Accuracy	F1-score	AUC
Training set	0.7661	0.7623	0.8342
Test set	0.65	0.65	0.7217
MIMIC	0.748	0.7386	0.814
Hospital	0.8075	0.8153	0.8748
MIMIC+ Hospital	0.7443	0.7421	0.8129

#### (3) Ablation study.

The third experimental part involves ablation study to assess the contribution of each modality and verify the effectiveness of the proposed multimodal data structure for sepsis clinical records. The textual and numerical modality datasets are derived by partitioning the multimodal dataset. The textual model employs the BioBERT module with a classification head, and the numerical model employs the BPNN module with a classification head. To ensure comparability, training settings are kept consistent with those used for the corresponding modules in the proposed BioBERT-BPNN multimodal model.

**Fig 7:**
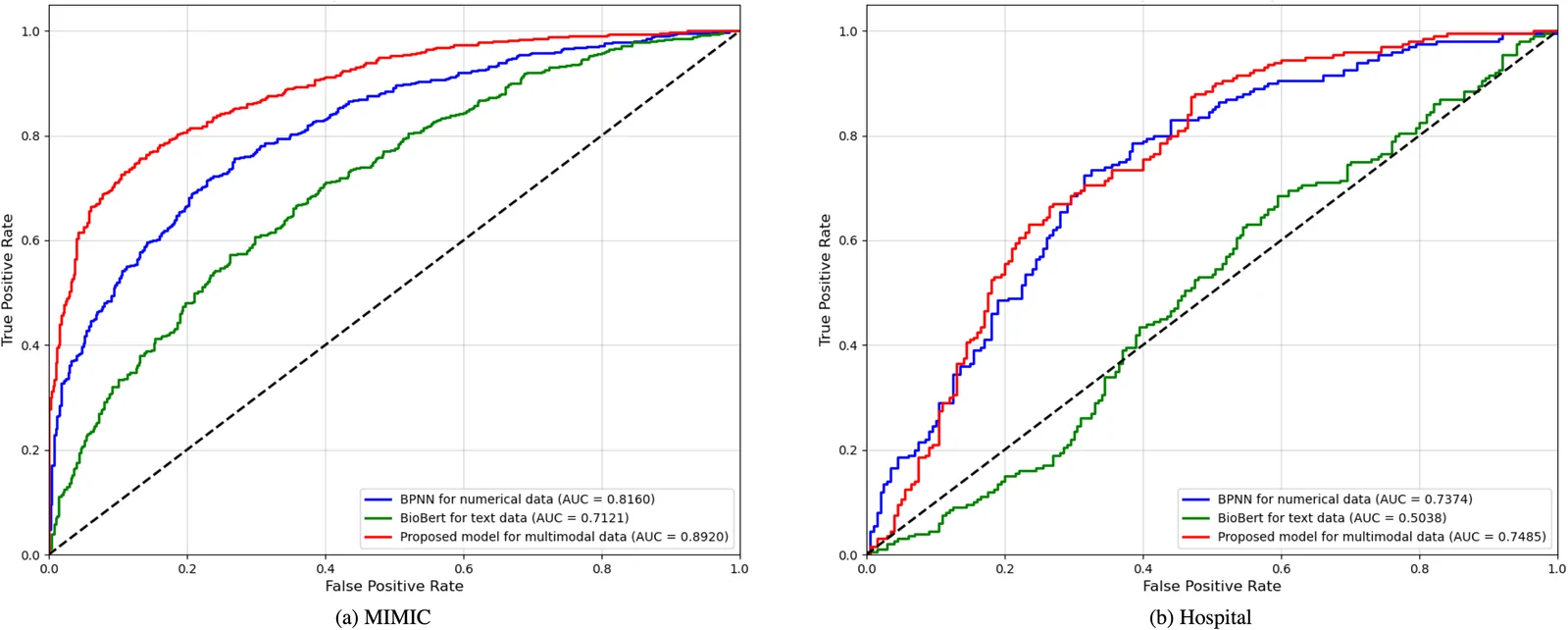
AUC-ROC curve comparison of modality-specific models under single-center validation on (a) the MIMIC dataset and (b) the Hospital dataset.

For the ablation study under single-center validation, the modality-specific models are trained on the MIMIC-training set and evaluated for early recognition of SA-AKI using both the MIMIC and the Hospital datasets. The corresponding results are summarized in Table 4 and Fig 7.

**Table 4:** Contribution of each modality under single-center validation.

Modality	Dataset	Accuracy	F1-score	AUC
Textual modality	MIMIC	0.6470	0.6217	0.7121
	Hospital	0.4925	0.4047	0.5038
Numerical modality	MIMIC	0.7240	0.6885	0.8160
	Hospital	0.6875	0.6803	0.7374
Multimodal	MIMIC	0.8110	0.7983	0.8920
	Hospital	0.6925	0.6886	0.7485

**Fig 8:**
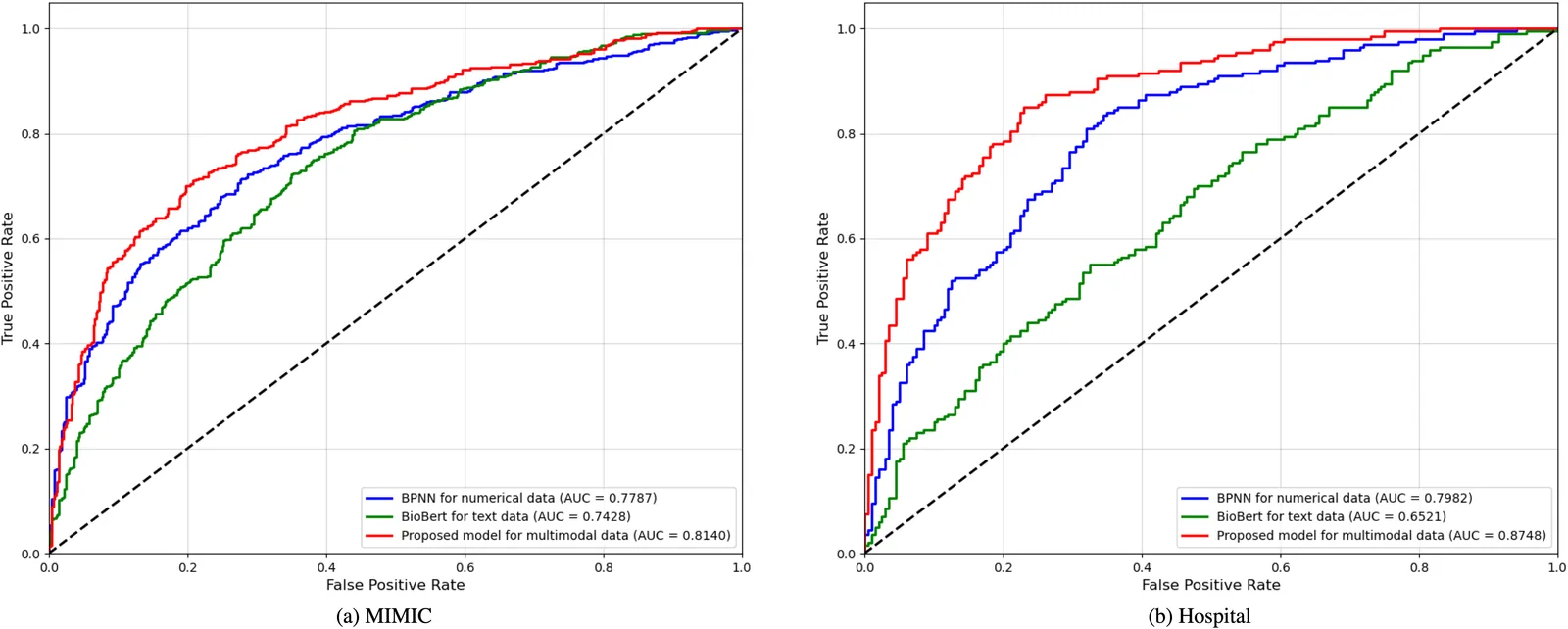
AUC-ROC curve comparison of modality-specific models under multi-center validation on (a) the MIMIC dataset and (b) the Hospital dataset.

For the ablation study under multi-center validation, each modality-specific model is trained and evaluated on the combined dataset of MIMIC and hospital and also evaluated separately on the MIMIC and Hospital datasets. The results are presented in Table and Fig 8.

As shown in the quantitative results (Tables 2-5) and qualitative results (Figs 7 and 8), ablation results consistently indicate that either modality alone could provide limited classification ability, and the multimodal model significantly outperforms single-modality models across Accuracy, F1-score, and AUC. Meanwhile, the performance also varies between two single-modality models, with the numerical modality model outperforming the textual modality model. These indicators can provide feature extractions with greater sensitivity and timeliness for the early recognition of SA-AKI. Therefore, these ablation results confirm the effectiveness of the designed multimodal data structure for sepsis clinical records, and demonstrate that the proposed BioBERT-BPNN multimodal model is able to capture complementary information from textual and numerical modalities effectively.

**Table 5:** Contribution of each modality under multi-center validation.

Modality	Dataset	Accuracy	F1-score	AUC
Textual modality	MIMIC	0.656	0.5914	0.7428
	Hospital	0.595	0.601	0.6521
	MIMIC+ Hospital	0.6386	0.5946	0.7133
Numerical modality	MIMIC	0.706	0.6541	0.7787
	Hospital	0.7175	0.7139	0.7982
	MIMIC+ Hospital	0.7064	0.6709	0.7692
Multimodal	MIMIC	0.7480	0.7386	0.8140
	Hospital	0.8075	0.8153	0.8748
	MIMIC+ Hospital	0.7443	0.7421	0.8129

## 5. Discussion

The pathogenesis of SA-AKI is highly complex, involving multiple pathological processes such as inflammatory responses, circulatory disturbances, infection control, and therapeutic interventions. Traditional statistical approaches rely heavily on empirical judgment and exhibit limitations in both sensitivity and specificity of research. Although risk scoring systems, furosemide stress tests, and various biomarkers have been introduced in recent years to facilitate early recognition of SA-AKI, their accuracy and clinical practicality remain insufficient, and they still fail to fully meet clinical needs [30].

With the rapid development of artificial intelligence and big data technologies, machine learning and deep learning have been increasingly applied to the study of SA-AKI. From a clinical perspective, accurate identification of high-risk patients is essential, as it facilitates timely renal protection, supports optimized treatment decision-making, and promotes efficient allocation of medical resources. The BioBERT-BPNN multimodal model proposed in this study integrates heterogeneous multi-source data from dual-center datasets to uncover potential nonlinear relationships and high-dimensional feature interactions. This approach demonstrates advantages in early recognition of SA-AKI.

It is worth clarifying the relationship among the accuracy values reported for the training set, the test set, and the full dataset. The full-dataset metrics (e.g., MIMIC as a whole) necessarily include the training samples on which the model was fitted, so they partly reflect in-sample fit rather than pure generalization performance; the test set and, more importantly, the external Hospital dataset provide the genuine measures of the model’s generalizability. Under single-center validation, the MIMIC-test accuracy (0.7550) was therefore lower than the MIMIC-training accuracy (0.8225), consistent with an expected, moderate degree of overfitting rather than a data or modeling error. Notably, the model’s accuracy on the external Hospital dataset (0.6925) was lower still than on MIMIC-test, which highlights a cross-center performance gap. This gap is plausibly attributable to differences in population characteristics, disease severity distribution, and clinical documentation practices between the U.S.-based MIMIC-IV cohort and the Chinese hospital cohort. The ablation results reinforce this interpretation: the textual-modality model, which depends heavily on BioBERT’s semantic representations learned predominantly from English-language biomedical corpora, showed a marked performance drop on the Hospital dataset (Accuracy 0.4925, close to chance level) compared with MIMIC (Accuracy 0.6470), whereas the numerical-modality model degraded far less (0.7240 on MIMIC vs. 0.6875 on Hospital). This asymmetry suggests that the textual modality is more sensitive to cross-linguistic and cross-institutional documentation differences, while structured numerical variables generalize more robustly across centers. The multimodal fusion model mitigates this gap by relying more heavily on the numerical modality’s cross-center robustness while still leveraging textual semantics when informative, which explains why the fused model’s cross-center performance loss (MIMIC 0.8110 vs. Hospital 0.6925) is proportionally smaller than that of the text-only model. These findings are clinically meaningful: they indicate that structured numerical indicators remain the more transferable signal for early SA-AKI recognition across institutions and languages, while free-text information adds value primarily within a single documentation system, a consideration relevant to future multicenter deployment of the model.

Despite encouraging results, several limitations should be acknowledged. First, the proposed model is developed using sepsis clinical data from the MIMIC public database and the EMR system of Shandong Public Health Clinical Center. Although dual-center data are used, the sample representativeness is still limited, and the lack of large-scale external validation constrains the model’s generalizability across diverse clinical populations. Second, the intrinsic “black-box” characteristics of deep learning models result in poor interpretability, which will impact clinicians’ confidence and acceptance of the model in clinical practice.

Therefore, future research will focus on three directions. First, greater collaboration with regional medical institutions is needed to facilitate data sharing under strict privacy safeguards, thereby supporting the development of multicenter epidemiological research models that improve representativeness and clinical applicability to the local population. Second, it is necessary to explore interpretable approaches that integrate artificial intelligence with traditional statistical methods, thereby improving model transparency and enhancing trust among clinicians and patients. Third, dynamic prediction tools leveraging real-time monitoring data could be designed to provide continuous risk assessment. Beyond regional collaboration within China, future work should also pursue larger and more representative samples, including data from geographically and ethnically diverse populations, to enable broader external validation and to determine whether the proposed model can be generalized for application across different healthcare systems worldwide.

## 6. Conclusion

This study develops a BioBERT-BPNN multimodal model for the early recognition of SA-AKI by integrating structured textual and numerical clinical data from dual centers. The model demonstrates stable predictive performance on the MIMIC dataset and maintains strong generalizability on the external Hospital dataset. Incorporating local hospital data further improves adaptability to the local target population, underscoring its clinical applicability. Ablation experiments confirm that combining textual and numerical modalities effectively captures complementary information and enhances predictive accuracy. Despite limited external validation and model interpretability, this work highlights the potential of artificial intelligence with complex clinical data to support timely intervention, optimize decision-making, and improve outcomes in sepsis patients at risk of SA-AKI.

## Supporting information

S1 Table Baseline characteristics of the selected sepsis cases from the MIMIC-IV database and Shandong Public Health Clinical Center. (DOCX)

## References

[1] KDIGO Acute Kidney Injury Work Group. KDIGO clinical practice guideline for acute kidney injury. Kidney International Supplements. 2012;2(1):1–138. doi: 10.1038/kisup.2012.1

[2] Hoste EAJ, Kellum JA, Selby NM, Zarbock A, Palevsky PM, Bagshaw SM, et al. Global epidemiology and outcomes of acute kidney injury. Nat Rev Nephrol. 2018;14(10):607–25. doi: 10.1038/s41581-018-0052-0 30135570

[3] Rhodes A, Evans LE, Alhazzani W, Levy MM, Antonelli M, Ferrer R, et al. Surviving sepsis campaign: international guidelines for management of sepsis and septic shock: 2016. Intensive Care Med. 2017;43(3):304–77. doi: 10.1007/s00134-017-4683-6 28101605

[4] Hoste EAJ, Bagshaw SM, Bellomo R, Cely CM, Colman R, Cruz DN, et al. Epidemiology of acute kidney injury in critically ill patients: the multinational AKI-EPI study. Intensive Care Med. 2015;41(8):1411–23. doi: 10.1007/s00134-015-3934-7 26162677

[5] Peerapornratana S, Manrique-Caballero CL, Gómez H, Kellum JA. Acute kidney injury from sepsis: current concepts, epidemiology, pathophysiology, prevention and treatment. Kidney Int. 2019;96(5):1083–99. doi: 10.1016/j.kint.2019.05.026 31443997 PMC6920048

[6] Bellomo R, Kellum JA, Ronco C. Acute kidney injury. Lancet. 2012;380(9843):756–66. doi: 10.1016/S0140-6736(11)61454-2 22617274

[7] Nian W, Tao W, Zhang H. Review of research progress in sepsis-associated acute kidney injury. Front Mol Biosci. 2025;12:1603392. doi: 10.3389/fmolb.2025.1603392 40718792 PMC12289498

[8] Yang H, Chen Y, He J, Li Y, Feng Y. Advances in the diagnosis of early biomarkers for acute kidney injury: a literature review. BMC Nephrol. 2025;26(1):115. doi: 10.1186/s12882-025-04040-3 40045274 PMC11884078

[9] Song X, Liu X, Liu F, Wang C. Comparison of machine learning and logistic regression models in predicting acute kidney injury: A systematic review and meta-analysis. Int J Med Inform. 2021;151:104484. doi: 10.1016/j.ijmedinf.2021.104484 33991886

[10] Li J, Zhu M, Yan L. Predictive models of sepsis-associated acute kidney injury based on machine learning: a scoping review. Ren Fail. 2024;46(2):2380748. doi: 10.1080/0886022X.2024.2380748 39082758 PMC11293267

[11] Zhou J, Bai Y, Wang X, Yang J, Fu P, Cai D, et al. A simple risk score for prediction of sepsis associated-acute kidney injury in critically ill patients. J Nephrol. 2019;32(6):947–56. doi: 10.1007/s40620-019-00625-y 31313123

[12] Deng F, Peng M, Li J, Chen Y, Zhang B, Zhao S. Nomogram to predict the risk of septic acute kidney injury in the first 24 h of admission: an analysis of intensive care unit data. Ren Fail. 2020;42(1):428–36. doi: 10.1080/0886022X.2020.1761832 32401139 PMC7269058

[13] Fan C, Ding X, Song Y. A new prediction model for acute kidney injury in patients with sepsis. Ann Palliat Med. 2021;10(2):1772–8. doi: 10.21037/apm-20-1117 33353355

[14] Ma J, Deng Y, Lao H, Ouyang X, Liang S, Wang Y, et al. A nomogram incorporating functional and tubular damage biomarkers to predict the risk of acute kidney injury for septic patients. BMC Nephrol. 2021;22(1):176. doi: 10.1186/s12882-021-02388-w 33985459 PMC8120900

[15] Xie Y, Zhang Y, Tian R. A prediction model of sepsis-associated acute kidney injury based on antithrombin III. Clinical and Experimental Med. 2021;21(1):89–100. doi: 10.1007/s10238-020-00656-x 32865720

[16] Yang S, Su T, Huang L, Feng L-H, Liao T. A novel risk-predicted nomogram for sepsis associated-acute kidney injury among critically ill patients. BMC Nephrol. 2021;22(1):173. doi: 10.1186/s12882-021-02379-x 33971853 PMC8111773

[17] Zhang L, Wang Z, Zhou Z, Li S, Huang T, Yin H, et al. Developing an ensemble machine learning model for early prediction of sepsis-associated acute kidney injury. iScience. 2022;25(9):104932. doi: 10.1016/j.isci.2022.104932 36060071 PMC9429796

[18] Jiang W, Zhang Y, Weng J, Song L, Liu S, Li X, et al. Explainable Machine Learning Model for Predicting Persistent Sepsis-Associated Acute Kidney Injury: Development and Validation Study. J Med Internet Res. 2025;27:e62932. doi: 10.2196/62932 40200699 PMC12070005

[19] Johnson AEW, Pollard TJ, Shen L, Lehman L-WH, Feng M, Ghassemi M, et al. MIMIC-III, a freely accessible critical care database. Sci Data. 2016;3:160035. doi: 10.1038/sdata.2016.35 27219127 PMC4878278

[20] Johnson AEW, Bulgarelli L, Shen L, Gayles A, Shammout A, Horng S, et al. MIMIC-IV, a freely accessible electronic health record dataset. Sci Data. 2023;10(1):1. doi: 10.1038/s41597-022-01899-x 36596836 PMC9810617

[21] He J, Lin J, Duan M. Application of machine learning to predict acute kidney disease in patients with sepsis associated acute kidney injury. Front Med (Lausanne). 2021;8:792974. doi: 10.3389/fmed.2021.792974 34957162 PMC8703139

[22] Mao C, Yao L, Luo Y. A Pre-trained Clinical Language Model for Acute Kidney Injury. In: 2020 IEEE International Conference on Healthcare Informatics (ICHI), 2020. 1–2. doi: 10.1109/ichi48887.2020.9374312

[23] Suh SH, Kim CS, Choi JS, Bae EH, Ma SK, Kim SW. Acute kidney injury in patients with sepsis and septic shock: risk factors and clinical outcomes. Yonsei Med J. 2013;54(4):965–72. doi: 10.3349/ymj.2013.54.4.965 23709433 PMC3663224

[24] Zarbock A, Nadim MK, Pickkers P, Gomez H, Bell S, Joannidis M, et al. Sepsis-associated acute kidney injury: consensus report of the 28th Acute Disease Quality Initiative workgroup. Nat Rev Nephrol. 2023;19(6):401–17. doi: 10.1038/s41581-023-00683-3 36823168

[25] Jiang Y-J, Xi X-M, Jia H-M, Zheng X, Wang M-P, Li W, et al. Risk factors, clinical features and outcome of new-onset acute kidney injury among critically ill patients: a database analysis based on prospective cohort study. BMC Nephrol. 2021;22(1):289. doi: 10.1186/s12882-021-02503-x 34433442 PMC8390222

[26] Liu J, Xie H, Ye Z, Li F, Wang L. Rates, predictors, and mortality of sepsis-associated acute kidney injury: a systematic review and meta-analysis. BMC Nephrol. 2020;21(1):318. doi: 10.1186/s12882-020-01974-8 32736541 PMC7393862

[27] Devlin J, Chang MW, Lee K. BERT: Pre-training of Deep Bidirectional Transformers for Language Understanding. In: Conference on the North American Chapter of the Association for Computational Linguistics: Human Language Technologies (NAACL‑HLT). 2019;4171–86. doi: 10.18653/v1/N19-1423

[28] Lee J, Yoon W, Kim S, Kim D, Kim S, So CH, et al. BioBERT: a pre-trained biomedical language representation model for biomedical text mining. Bioinformatics. 2020;36(4):1234–40. doi: 10.1093/bioinformatics/btz682 31501885 PMC7703786

[29] Vaswani A, Shazeer N, Parmar N. Attention is all you need. Advances in Neural Information Processing Systems. 2017;30:5998–6008. doi: 10.48550/arXiv.1706.03762

[30] Birkelo BC, Pannu N, Siew ED. Overview of diagnostic criteria and epidemiology of acute kidney injury and acute kidney disease in the critically Ill patient. Clin J Am Soc Nephrol. 2022;17(5):717–35. doi: 10.2215/CJN.14181021 35292532 PMC9269585

