# Supplementary material for: Early recognition of sepsis-associated acute kidney injury based on BioBERT-BPNN multimodal clinical data analysis
Source: PLoS One. 2026 Sep 11;21(9):e0357602. doi: 10.1371/journal.pone.0357602 (PMC13567760; doi:10.1371/journal.pone.0357602)
Supplement: Supplementary file 1 [file pone.0357602.s001.docx]

**S1 Table**

1 Baseline characteristics of the selected sepsis cases from the MIMIC-IV database

The baseline data of 1,000 sepsis cases in MIMIC-IV are shown below, with continuous variables presented as mean ± standard deviation.

MIMIC-IV cohort (n=1,000)

(a) Demographic characteristics

| Variables | Total(n=1000) | SA-AKI (n=500) | Non-SA-AKI(n=500) |
| --- | --- | --- | --- |
| Male (n(%)) | 455(45.50%) | 207(41.40%) | 248(49.60%) |
| Age (yeas) | 63.75±15.59 | 63.68±15.65 | 63.81±15.52 |
| BMI (kg/m²) | 26.92±4.98 | 26.88±4.98 | 26.96±4.97 |

(b) Disease severity scores

| Variables | Total(n=1000) | SA-AKI (n=500) | Non-SA-AKI(n=500) |
| --- | --- | --- | --- |
| SOFA | 6.27±3.90 | 7.23±3.94 | 5.30±3.61 |
| SAPS II | 20.15±5.86 | 21.56±5.53 | 18.74±5.83 |
| GCS | 13.64±2.73 | 13.76±2.63 | 13.52±2.81 |

(c) Comorbidities

| Variables | Total(n=1000) | SA-AKI (n=500) | Non-SA-AKI(n=500) |
| --- | --- | --- | --- |
| Coronary heart disease (n(%)) | 189(18.90%) | 90(18.00%) | 99(19.80%) |
| COPD (n(%)) | 29(2.90%) | 11(2.20%) | 18(3.60%) |
| Hypertension (n(%)) | 363(36.30%) | 174(34.80%) | 189(37.80%) |
| Diabetes (n(%)) | 301(30.10%) | 159(31.80%) | 142(28.40%) |
| Cerebral infarction (n(%)) | 90(9.00%) | 44(8.80%) | 46(9.20%) |
| Chronic renal failure (n(%)) | 193(19.30%) | 116(23.20%) | 77(15.40%) |
| Liver disease (n(%)) | 247(24.70%) | 137(27.40%) | 110(22.00%) |
| Trauma (n(%)) | 75(7.50%) | 34(6.80%) | 41(8.20%) |
| ARDS (n(%)) | 516(51.60%) | 277(55.40%) | 239(47.80%) |

(d) Treatment interventions

| Variables | Total(n=1000) | SA-AKI (n=500) | Non-SA-AKI(n=500) |
| --- | --- | --- | --- |
| Invasive mechanical ventilation (n(%)) | 560(56.00%) | 329(65.80%) | 231(46.20%) |
| Vasopressors (n(%)) | 619(61.90%) | 328(65.60%) | 291(58.20%) |

(e) Laboratory examinations

| Variables | Total(n=1000) | SA-AKI (n=500) | Non-SA-AKI(n=500) |
| --- | --- | --- | --- |
| Red blood cells (*10^12^/L) | 3.85±0.76 | 3.83±0.75 | 3.86±0.48 |
| Hemoglobin (g/L) | 11.60±2.25 | 11.56±2.26 | 11.63±2.24 |
| White blood cells (*10^9^/L) | 11.60±2.25 | 13.09±8.71 | 10.98±7.28 |
| Platelets (*10^9^/L) | 245.59±140.87 | 247.62±150.77 | 243.57±130.20 |
| ALT (U/L) | 88.88±407.49 | 123.62±550.47 | 54.13±163.29 |
| Bilirubin (mg/dL) | 1.62±3.53 | 2.10±4.50 | 1.15±2.03 |
| Albumin (g/L) | 3.08±0.73 | 3.02±0.73 | 3.15±0.73 |
| Creatinine (mg/dL) | 1.60±1.69 | 2.04±1.92 | 1.15±1.28 |
| BUN (mg/dL) | 30.40±25.13 | 37.10±28.79 | 23.70±18.56 |
| Blood glucose (mg/dL) | 146.09±88.38 | 155.88±109.84 | 136.30±57.99 |
| Prothrombin Time (s) | 16.12±7.85 | 16.53±7.70 | 15.72±7.98 |
| Ph | 7.36±0.11 | 7.35±0.12 | 7.37±0.11 |
| Potassium (mmol/L) | 4.30±0.90 | 4.40±1.05 | 4.20±0.72 |
| Sodium (mmol/L) | 137.78±5.31 | 137.93±5.66 | 137.63±4.93 |
| Calcium (mg/dL) | 8.46±1.02 | 8.45±1.05 | 8.74±0.98 |
| Lactate (mmol/L) | 2.74±2.32 | 3.03±2.58 | 2.45±1.98 |

2 Baseline characteristics of the selected sepsis cases from Shandong Public Health Clinical Center

The baseline data of 400 sepsis cases in Shandong Public Health Clinical Center EMR system are shown below, with continuous variables presented as mean ± standard deviation.

Shandong Public Health Clinical Center cohort (n=400)

(a) Demographic characteristics

| Variables | Total(n=400) | SA-AKI (n=200) | Non-SA-AKI(n=200) |
| --- | --- | --- | --- |
| Male (n(%)) | 164(41.00%) | 77(38.50%) | 87(43.50%) |
| Age (yeas) | 64.45±9.75 | 63.76±9.49 | 65.15±9.97 |
| BMI (kg/m²) | 25.33±3.34 | 25.39±3.36 | 25.27±3.33 |

(b) Disease severity scores

| Variables | Total(n=400) | SA-AKI (n=200) | Non-SA-AKI(n=200) |
| --- | --- | --- | --- |
| SOFA | 10.88±3.66 | 11.42±3.70 | 10.33±3.54 |
| SAPS II | 28.20±6.13 | 29.27±6.35 | 27.12±5.69 |
| GCS | 12.07±2.02 | 12.01±2.16 | 12.14±1.86 |

(c) Comorbidities

| Variables | Total(n=400) | SA-AKI (n=200) | Non-SA-AKI(n=200) |
| --- | --- | --- | --- |
| Coronary heart disease (n(%)) | 103(25.75%) | 53(26.50%) | 50(25.00%) |
| COPD (n(%)) | 110(27.50%) | 50(25.00%) | 60(30.00%) |
| Hypertension (n(%)) | 167(41.75%) | 78(39.00%) | 89(44.50%) |
| Diabetes (n(%)) | 101(25.25%) | 59(29.50%) | 42(21.00%) |
| Cerebral infarction (n(%)) | 30(7.50%) | 15(7.50%) | 15(7.50%) |
| Chronic renal failure (n(%)) | 60(15.00%) | 40(20.00%) | 20(10.00%) |
| Liver disease (n(%)) | 99(24.75%) | 57(28.50%) | 42(21.00%) |
| Trauma (n(%)) | 19(4.75%) | 13(6.50%) | 6(3.00%) |
| ARDS (n(%)) | 176(44.00%) | 94(47.00%) | 82(41.00%) |

(d) Treatment interventions

| Variables | Total(n=400) | SA-AKI (n=200) | Non-SA-AKI(n=200) |
| --- | --- | --- | --- |
| Invasive mechanical ventilation (n(%)) | 157(39.25%) | 72(36.00%) | 85(42.50%) |
| Vasopressors (n(%)) | 240(60.00%) | 123(61.50%) | 117(58.50%) |

(e) Laboratory examinations

| Variables | Total(n=400) | SA-AKI (n=200) | Non-SA-AKI(n=200) |
| --- | --- | --- | --- |
| Red blood cells (*10^9^/L) | 3.70±0.61 | 3.66±0.57 | 3.74±0.64 |
| Hemoglobin (g/L) | 11.10±2.39 | 10.96±2.37 | 11.24±22.40 |
| Leukocyte (*10^9^/L) | 15.29±4.85 | 15.38±5.01 | 15.21±4.69 |
| Thrombocyte (*10^9^/L) | 127.00±38.39 | 123.44±40.75 | 130.57±30.50 |
| ALT (U/L) | 45.13±31.20 | 46.42±33.18 | 43.83±29.03 |
| Bilirubin (μmol/L) | 5.07±4.20 | 5.43±4.51 | 4.71±3.82 |
| Albumin (g/L) | 2.90±0.40 | 2.88±0.39 | 2.92±0.41 |
| Creatinine (mg/dL) | 1.60±0.43 | 1.88±0.29 | 1.31±0.35 |
| BUN (mmol/L) | 31.33±18.98 | 33.24±19.12 | 29.41±18.64 |
| Blood Glucose (mg/dL) | 174.47±60.29 | 177.46±56.33 | 171.54±63.86 |
| Prothrombin Time (s) | 15.08±1.89 | 15.14±1.81 | 15.02±1.96 |
| Ph | 7.40±0.07 | 7.40±0.07 | 7.40±0.07 |
| Sodium (mmol/L) | 140.16±4.76 | 140.47±4.69 | 139.85±4.81 |
| Potassium (mmol/L) | 3.99±0.50 | 4.00±0.50 | 3.98±0.50 |
| Calcium (mg/dL) | 7.96±1.08 | 8.00±1.07 | 7.93±1.10 |
| Lactate (mmol/L) | 3.12±2.94 | 3.37±3.29 | 2.87±2.52 |
