# Supplementary material for: Early recognition of sepsis-associated acute kidney injury based on BioBERT-BPNN multimodal clinical data analysis
Source: PLoS One. 2026 Sep 11;21(9):e0357602. doi: 10.1371/journal.pone.0357602 (PMC13567760; doi:10.1371/journal.pone.0357602)
Supplement: Supplementary file 2 [file pone.0357602.s002.docx]

**Response to Technical Check**

Manuscript ID: PONE-D-26-29602

Manuscript title: Early recognition of sepsis-associated acute kidney injury based on BioBERT-BPNN multimodal clinical data analysis

Dear Editorial Office,

Thank you for your message and for checking our revised submission.

We have revised the Methods section according to your request. Specifically, we have added the dates when the data were accessed and extracted for research purposes in DD/MM/YYYY format.

The added statement is as follows:

“The MIMIC-IV data were accessed for research purposes through PhysioNet from 01/01/2026 to 01/03/2026. The de-identified EMR data from Shandong Public Health Clinical Center were accessed and extracted for research purposes from 01/01/2026 to 01/03/2026.”

We understand that this information refers to the period during which the study data were accessed or extracted for research purposes, rather than the period during which participants were admitted to the hospital.

Thank you for your consideration.

Sincerely,

Yufeng Chen
