# Supplementary material for: Early recognition of sepsis-associated acute kidney injury based on BioBERT-BPNN multimodal clinical data analysis
Source: PLoS One. 2026 Sep 11;21(9):e0357602. doi: 10.1371/journal.pone.0357602 (PMC13567760; doi:10.1371/journal.pone.0357602)
Supplement: Supplementary file 3 [file pone.0357602.s003.docx]

**Response to Reviewers**

**Manuscript: PONE-D-26-29602**

**Title: Early Recognition of Sepsis-Associated Acute Kidney Injury Based on BioBERT-BPNN Multimodal Clinical Data Analysis**

Dear Dr. Al-Nimer and Reviewers,

We thank the Academic Editor and both reviewers for their careful evaluation of our manuscript and for the constructive comments. We have revised the manuscript accordingly and provide a point-by-point response below. All changes are highlighted in the marked-up version of the manuscript.

**A. Response to Academic Editor — Journal Requirements**

***1. Manuscript formatting and file naming***

Response: We have reviewed the PLOS ONE formatting templates (main body and title/authors/affiliations) and revised the manuscript and file names accordingly to comply with PLOS ONE style requirements.

***2. Code sharing***

Response: We agree that the author-generated code underpinning the findings of this study should be made available. The code used for data preprocessing, feature construction, and model development (BioBERT-BPNN multimodal model) has been deposited in a public GitHub repository: https://github.com/cyf19931021/BioBERT-BPNN-SAAKI. This repository will remain publicly accessible without restriction upon publication. A corresponding Code Availability statement has been added to the manuscript (Data availability section).

***3. Ethics statement location***

Response: Thank you for pointing this out. We have removed the duplicate ethics approval details (approval number and waiver of informed consent) from the Data availability section at the end of the manuscript, replacing them with a brief cross-reference to the Methods section. The full ethics statement now appears only once, in the Methods section (3.1 Data source).

***4. Data availability***

Response: We confirm that our Data Availability statement follows the required format. The MIMIC-IV data are publicly available upon completion of the standard data use agreement and training (https://mimic.mit.edu/). The hospital EMR data cannot be made publicly available due to patient privacy and institutional ethical restrictions; qualified researchers may request access from the corresponding author, subject to approval by the Ethics Committee of Shandong Public Health Clinical Center, as stated in the manuscript. We respectfully request that this restriction be recognized as an approved exemption given the sensitive nature of the hospital clinical data.

***5. LaTeX template***

Response: This manuscript was prepared and submitted in Microsoft Word format, following PLOS ONE's Word-based formatting guidelines rather than the LaTeX template, as our submission does not involve LaTeX-specific typesetting. We would appreciate the Editor's confirmation that a Word submission remains acceptable; if a LaTeX version is required, we are glad to provide one upon request.

***6. Title consistency***

Response: We have confirmed that the title in the online submission system is identical to the title on the manuscript ("Early Recognition of Sepsis-Associated Acute Kidney Injury Based on BioBERT-BPNN Multimodal Clinical Data Analysis").

***7. Abstract consistency***

Response: We have confirmed that the abstract in the online submission system is identical to the abstract in the manuscript.

***8. Figures within the manuscript file***

Response: We have removed all 8 embedded figures from within the manuscript text, retaining only the figure legends in place at their original locations. All 8 figures have been re-exported in TIFF format at a resolution within PLOS ONE's required 300-600 DPI range and a minimum width of 789 pixels, and have been uploaded separately as individual submission items, replacing the previous PNG versions, as required.

***9. Supporting Information captions***

Response: We have added a caption for our Supporting Information file (S1 Table: Baseline characteristics of the selected sepsis cases from the MIMIC-IV database and Shandong Public Health Clinical Center) at the end of the manuscript, under a new "Supporting information" heading. The in-text citation, previously referring to "the Appendix," has been updated to cite "S1 Table" accordingly (Methods, Section 3.2).

***10. Reviewer-suggested citations***

Response: Neither reviewer recommended specific additional citations; therefore, this item does not apply to our revision.

***11. Reference list accuracy***

Response: We have reviewed the full reference list and confirm that it is complete and accurate, and that none of the cited works have been retracted.

**B. Response to Reviewer #1**

We sincerely thank Reviewer #1 for the positive and encouraging evaluation of our manuscript, and for recognizing the methodological rigor involved in integrating and analyzing the dual-center datasets.

We fully agree with the reviewer's comment regarding the limitations related to sample size and the lack of geographically and ethnically diverse external validation. We have further emphasized this point in the Discussion and Conclusion sections, and have outlined our plan to pursue broader multicenter collaboration — including institutions from different regions — in future work to strengthen the external validity and generalizability of the proposed model (see revised Discussion, "future research" paragraph).

**C. Response to Reviewer #2**

***Comment 1: "Please elaborate how the accuracy of training set and testing set were lower than the actual MIMIC and hospital on the validation."***

Response: We thank the reviewer for raising this important point, which we agree required clearer explanation in the original submission.

The apparent discrepancy arises from the composition of each reported dataset. The “MIMIC” and “Hospital” rows in Tables 2–5 represent performance on the entire respective dataset, which necessarily includes the samples used for model training; these full-dataset metrics therefore partly reflect in-sample fit rather than purely independent generalization performance. In contrast, the “MIMIC-test” and “Test set” rows report performance on held-out samples never seen during training, which constitute the genuine measure of the model's generalization ability.

Under single-center validation (Table 2), the MIMIC-test accuracy (0.7550) is lower than the MIMIC-training accuracy (0.8225) and the full MIMIC accuracy (0.8110), consistent with an expected, moderate degree of overfitting typical of deep learning models trained on data of this scale — this is not indicative of a data-processing or modeling error. We have added an explanatory paragraph to the Discussion section clarifying this relationship (see revised manuscript, Discussion, second added paragraph).

We further note that the external Hospital dataset accuracy (0.6925) is lower still than the MIMIC-test accuracy, reflecting a genuine cross-center performance gap rather than an artifact of data splitting; this gap and its plausible explanations are addressed in our response to Comment 2 below.

***Comment 2: "Discussion can be elaborated more as to how and why the results obtained are more relevant and of importance and plausible explanations."***

Response: We thank the reviewer for this valuable suggestion. We have substantially expanded the Discussion section to provide a more detailed, mechanism-based interpretation of our results, rather than a general summary. Specifically, we now discuss:

(1) Why the cross-center performance gap (MIMIC vs. Hospital) is plausibly attributable to differences in population characteristics, disease severity distribution, and clinical documentation practices between the U.S.-based MIMIC-IV cohort and the Chinese hospital cohort;

(2) Why this gap is asymmetric across modalities: the ablation results show that the textual-modality model's accuracy drops sharply on the Hospital dataset (0.4925, close to chance level) compared with MIMIC (0.6470), while the numerical-modality model degrades much less (0.7240 on MIMIC vs. 0.6875 on Hospital). We attribute this to BioBERT's semantic representations being learned predominantly from English-language biomedical corpora, making the textual modality more sensitive to cross-linguistic and cross-institutional documentation differences, whereas structured numerical variables are inherently more transferable across centers;

(3) Why the multimodal fusion model is more robust than the text-only model in cross-center performance: by combining both modalities, the fused model can rely more heavily on the numerical modality's cross-center robustness while still benefiting from textual semantics when informative, resulting in a proportionally smaller performance loss (MIMIC 0.8110 vs. Hospital 0.6925) than the text-only model alone;

(4) The clinical relevance of these findings: structured numerical indicators appear to be the more transferable and reliable signal for early SA-AKI recognition when a model is to be deployed across institutions or languages, while free-text information adds the most value within a single, consistent documentation system — a consideration directly relevant to future multicenter deployment of the proposed model.

This expanded discussion can be found in the newly added paragraph immediately following the description of the BioBERT-BPNN model's advantages, at the beginning of the Discussion section (see revised manuscript, highlighted text).

We thank the Academic Editor and both reviewers again for their time and valuable feedback, which has substantially strengthened the clarity and depth of our manuscript.

Sincerely,

Yufeng Chen, Hongyang Bian, Yuye Li (Corresponding Author)
