# Supplementary material for: Early recognition of sepsis-associated acute kidney injury based on BioBERT-BPNN multimodal clinical data analysis
Source: PLoS One. 2026 Sep 11;21(9):e0357602. doi: 10.1371/journal.pone.0357602 (PMC13567760; doi:10.1371/journal.pone.0357602)
Supplement: Peer review history

# Author comment: Author response to Decision Letter 0

**DOI:** 10.1371/journal.pone.0357602.r001

24 Jun 2026

Attachment Submitted filename: Response to Technical Check 2.docx

# Peer review: Decision Letter 0

**DOI:** 10.1371/journal.pone.0357602.r002

**Authors:** Marwan Al-Nimer

12 Aug 2026

Dear Dr. Li,

Thank you for submitting your manuscript to PLOS ONE. After careful consideration, we feel that it has merit but does not fully meet PLOS ONE’s publication criteria as it currently stands. Therefore, we invite you to submit a revised version of the manuscript that addresses the points raised during the review process.

==============================

**ACADEMIC EDITOR: Minor revision**

Please submit your revised manuscript by Sep 11 2026 11:59PM. If you will need more time than this to complete your revisions, please reply to this message or contact the journal office at plosone@plos.org. When you're ready to submit your revision, log on to https://www.editorialmanager.com/pone/ and select the 'Submissions Needing Revision' folder to locate your manuscript file.

- A letter that responds to each point raised by the academic editor and reviewer(s). You should upload this letter as a separate file labeled 'Response to Reviewers'.
- A marked-up copy of your manuscript that highlights changes made to the original version. You should upload this as a separate file labeled 'Revised Manuscript with Track Changes'.
- An unmarked version of your revised paper without tracked changes. You should upload this as a separate file labeled 'Manuscript'.

If you would like to make changes to your financial disclosure, please include your updated statement in your cover letter. Guidelines for resubmitting your figure files are available below the reviewer comments at the end of this letter.

If applicable, we recommend that you deposit your laboratory protocols in protocols.io to enhance the reproducibility of your results. Protocols.io assigns your protocol its own identifier (DOI) so that it can be cited independently in the future. For instructions see: https://journals.plos.org/plosone/s/submission-guidelines#loc-laboratory-protocols. Additionally, PLOS ONE offers an option for publishing peer-reviewed Lab Protocol articles, which describe protocols hosted on protocols.io. Read more information on sharing protocols at https://plos.org/protocols?utm_medium=editorial-email&utm_source=authorletters&utm_campaign=protocols.

As the corresponding author, your ORCID iD is verified in the submission system and will appear in the published article. PLOS supports the use of ORCID, and we encourage all coauthors to register for an ORCID iD and use it as well. Please encourage your coauthors to verify their ORCID iD within the submission system before final acceptance, as unverified ORCID iDs will not appear in the published article. *Only*  the individual author can complete the verification step; PLOS staff *cannot*  verify ORCID iDs on behalf of authors.

We look forward to receiving your revised manuscript.

Kind regards,

Marwan Salih Al-Nimer, MD, PhD

Academic Editor

PLOS One

Journal Requirements:

When submitting your revision, we need you to address these additional requirements.

1. Please ensure that your manuscript meets PLOS ONE's style requirements, including those for file naming. The PLOS ONE style templates can be found at

https://journals.plos.org/plosone/s/file?id=wjVg/PLOSOne_formatting_sample_main_body.pdf and https://journals.plos.org/plosone/s/file?id=ba62/PLOSOne_formatting_sample_title_authors_affiliations.pdf

2. Please note that PLOS One has specific guidelines on code sharing for submissions in which author-generated code underpins the findings in the manuscript. In these cases, we expect all author-generated code to be made available without restrictions upon publication of the work. Please review our guidelines at https://journals.plos.org/plosone/s/materials-and-software-sharing#loc-sharing-code and ensure that your code is shared in a way that follows best practice and facilitates reproducibility and reuse.

3. Your ethics statement should only appear in the Methods section of your manuscript. If your ethics statement is written in any section besides the Methods, please delete it from any other section.

4. In the online submission form, you indicated that he MIMIC-IV data used in this study are publicly available from the MIMIC-IV database at https://mimic.mit.edu/, subject to completion of the required training and data use agreement. The hospital electronic medical record data used in this study were obtained from Shandong Public Health Clinical Center and contain sensitive clinical information. Although the dataset was de-identified before analysis, public sharing is restricted by ethical and privacy regulations. Researchers who meet the criteria for access to confidential clinical data may request access from the corresponding author, Yuye Li, at catherine1696@163.com, subject to approval by Shandong Public Health Clinical Center and its Ethics Committee. The use of the hospital data was approved by the Ethics Committee of Shandong Public Health Clinical Center (Shandong University Affiliated Public Health Clinical Center) (Approval No.: GWLCZXEC-SOP-K-2025-63)

All PLOS journals now require all data underlying the findings described in their manuscript to be freely available to other researchers, either 1. In a public repository, 2. Within the manuscript itself, or 3. Uploaded as supplementary information.

This policy applies to all data except where public deposition would breach compliance with the protocol approved by your research ethics board. If your data cannot be made publicly available for ethical or legal reasons (e.g., public availability would compromise patient privacy), please explain your reasons on resubmission and your exemption request will be escalated for approval.

5. Please update your submission to use the PLOS LaTeX template. The template and more information on our requirements for LaTeX submissions can be found at http://journals.plos.org/plosone/s/latex.

6. Please amend either the title on the online submission form (via Edit Submission) or the title in the manuscript so that they are identical.

7. Please amend either the abstract on the online submission form (via Edit Submission) or the abstract in the manuscript so that they are identical.

8. Please remove your figures from within your manuscript file, leaving only the individual TIFF/EPS image files, uploaded separately. These will be automatically included in the reviewers’ PDF**.**

9. Please include captions for your Supporting Information files at the end of your manuscript, and update any in-text citations to match accordingly. Please see our Supporting Information guidelines for more information: http://journals.plos.org/plosone/s/supporting-information.

10. If the reviewer comments include a recommendation to cite specific previously published works, please review and evaluate these publications to determine whether they are relevant and should be cited. There is no requirement to cite these works unless the editor has indicated otherwise.

11. Please review your reference list to ensure that it is complete and correct. If you have cited papers that have been retracted, please include the rationale for doing so in the manuscript text, or remove these references and replace them with relevant current references. Any changes to the reference list should be mentioned in the rebuttal letter that accompanies your revised manuscript. If you need to cite a retracted article, indicate the article’s retracted status in the References list and also include a citation and full reference for the retraction notice.

[Note: HTML markup is below. Please do not edit.]

Reviewers' comments:

Reviewer's Responses to Questions

**Comments to the Author**

1. Is the manuscript technically sound, and do the data support the conclusions?

Reviewer #1: Yes

Reviewer #2: Yes

**********

2. Has the statistical analysis been performed appropriately and rigorously?

Reviewer #1: Yes

Reviewer #2: Yes

**********

3. Have the authors made all data underlying the findings in their manuscript fully available?

The PLOS Data policy

Reviewer #1: Yes

Reviewer #2: Yes

**********

4. Is the manuscript presented in an intelligible fashion and written in standard English?

Reviewer #1: Yes

Reviewer #2: Yes

**********

Reviewer #1: The manuscript entitled “Early Recognition of Sepsis-Associated Acute Kidney Injury Based on BioBERT-BPNN Multimodal Clinical Data Analysis,” by Yuye Li and colleagues from the Shandong Provincial Public Health Clinical Center (Shandong Provincial Chest Hospital), Jinan, Shandong, China, evaluates a multimodal artificial intelligence model (BioBERT-BPNN) for the analysis and early identification of sepsis-associated acute kidney injury.

The study demonstrates that the authors exercised considerable methodological rigor. Analyzing two large databases comprising distinct patient populations, while seeking to ensure the robustness of the findings, requires careful model development, disciplined data handling, and rigorous validation procedures. In my assessment, these requirements were appropriately addressed. The authors obtained interesting and clinically relevant results regarding accuracy, F1 score, and area under the receiver operating characteristic curve (AUC), as clearly presented in the tables and figures.

Nevertheless, as appropriately acknowledged by the authors, the study’s limitations still restrict the model’s practical applicability, including within China. Larger and more representative samples will be required to strengthen the external validity of the findings. Furthermore, incorporating data from geographically and ethnically diverse populations would allow broader external validation and help determine whether the proposed model can be applied globally.

These limitations, however, do not diminish the relevance of the research conducted by Yuye Li and colleagues. On the contrary, this study contributes to expanding the potential applications of artificial intelligence in medicine, particularly its capacity to analyze large and complex datasets containing millions of data points. Nevertheless, human expertise remains indispensable for selecting and supplying appropriate data, critically interpreting the analyses, and translating their findings into clinically meaningful knowledge that may ultimately improve patient care.

Reviewer #2: Please elaborate how the accuracy of training set and testing set were lower than the actual MIMIC and hospital on the validation?

Discussion can be elaborated more as to how and why the results obtained are more relevant and of importance and plausible explanations?

**********

what does this mean?). If published, this will include your full peer review and any attached files.

If you choose “no”, your identity will remain anonymous but your review may still be made public.

**Do you want your identity to be public for this peer review?**  For information about this choice, including consent withdrawal, please see our Privacy Policy

Reviewer #1: No

Reviewer #2: **Yes:** Hari Naga Garapati

**********

[NOTE: If reviewer comments were submitted as an attachment file, they will be attached to this email and accessible via the submission site. Please log into your account, locate the manuscript record, and check for the action link "View Attachments". If this link does not appear, there are no attachment files.]

To ensure your figures meet our technical requirements, please review our figure guidelines: https://journals.plos.org/plosone/s/figures

You may also use PLOS’s free figure tool, NAAS, to help you prepare publication quality figures: https://journals.plos.org/plosone/s/figures#loc-tools-for-figure-preparation.

NAAS will assess whether your figures meet our technical requirements by comparing each figure against our figure specifications.

# Author comment: Author response to Decision Letter 1

**DOI:** 10.1371/journal.pone.0357602.r003

17 Aug 2026

Response to Reviewers

Manuscript: PONE-D-26-29602

Title: Early Recognition of Sepsis-Associated Acute Kidney Injury Based on BioBERT-BPNN Multimodal Clinical Data Analysis

Dear Dr. Al-Nimer and Reviewers,

We thank the Academic Editor and both reviewers for their careful evaluation of our manuscript and for the constructive comments. We have revised the manuscript accordingly and provide a point-by-point response below. All changes are highlighted in the marked-up version of the manuscript.

A. Response to Academic Editor — Journal Requirements

1. Manuscript formatting and file naming

Response: We have reviewed the PLOS ONE formatting templates (main body and title/authors/affiliations) and revised the manuscript and file names accordingly to comply with PLOS ONE style requirements.

2. Code sharing

Response: We agree that the author-generated code underpinning the findings of this study should be made available. The code used for data preprocessing, feature construction, and model development (BioBERT-BPNN multimodal model) has been deposited in a public GitHub repository: https://github.com/cyf19931021/BioBERT-BPNN-SAAKI. This repository will remain publicly accessible without restriction upon publication. A corresponding Code Availability statement has been added to the manuscript (Data availability section).

3. Ethics statement location

Response: Thank you for pointing this out. We have removed the duplicate ethics approval details (approval number and waiver of informed consent) from the Data availability section at the end of the manuscript, replacing them with a brief cross-reference to the Methods section. The full ethics statement now appears only once, in the Methods section (3.1 Data source).

4. Data availability

Response: We confirm that our Data Availability statement follows the required format. The MIMIC-IV data are publicly available upon completion of the standard data use agreement and training (https://mimic.mit.edu/). The hospital EMR data cannot be made publicly available due to patient privacy and institutional ethical restrictions; qualified researchers may request access from the corresponding author, subject to approval by the Ethics Committee of Shandong Public Health Clinical Center, as stated in the manuscript. We respectfully request that this restriction be recognized as an approved exemption given the sensitive nature of the hospital clinical data.

5. LaTeX template

Response: This manuscript was prepared and submitted in Microsoft Word format, following PLOS ONE's Word-based formatting guidelines rather than the LaTeX template, as our submission does not involve LaTeX-specific typesetting. We would appreciate the Editor's confirmation that a Word submission remains acceptable; if a LaTeX version is required, we are glad to provide one upon request.

6. Title consistency

Response: We have confirmed that the title in the online submission system is identical to the title on the manuscript ("Early Recognition of Sepsis-Associated Acute Kidney Injury Based on BioBERT-BPNN Multimodal Clinical Data Analysis").

7. Abstract consistency

Response: We have confirmed that the abstract in the online submission system is identical to the abstract in the manuscript.

8. Figures within the manuscript file

Response: We have removed all 8 embedded figures from within the manuscript text, retaining only the figure legends in place at their original locations. All 8 figures have been re-exported in TIFF format at a resolution within PLOS ONE's required 300-600 DPI range and a minimum width of 789 pixels, and have been uploaded separately as individual submission items, replacing the previous PNG versions, as required.

9. Supporting Information captions

Response: We have added a caption for our Supporting Information file (S1 Table: Baseline characteristics of the selected sepsis cases from the MIMIC-IV database and Shandong Public Health Clinical Center) at the end of the manuscript, under a new "Supporting information" heading. The in-text citation, previously referring to "the Appendix," has been updated to cite "S1 Table" accordingly (Methods, Section 3.2).

10. Reviewer-suggested citations

Response: Neither reviewer recommended specific additional citations; therefore, this item does not apply to our revision.

11. Reference list accuracy

Response: We have reviewed the full reference list and confirm that it is complete and accurate, and that none of the cited works have been retracted.

B. Response to Reviewer #1

We sincerely thank Reviewer #1 for the positive and encouraging evaluation of our manuscript, and for recognizing the methodological rigor involved in integrating and analyzing the dual-center datasets.

We fully agree with the reviewer's comment regarding the limitations related to sample size and the lack of geographically and ethnically diverse external validation. We have further emphasized this point in the Discussion and Conclusion sections, and have outlined our plan to pursue broader multicenter collaboration — including institutions from different regions — in future work to strengthen the external validity and generalizability of the proposed model (see revised Discussion, "future research" paragraph).

C. Response to Reviewer #2

Comment 1: "Please elaborate how the accuracy of training set and testing set were lower than the actual MIMIC and hospital on the validation."

Response: We thank the reviewer for raising this important point, which we agree required clearer explanation in the original submission.

The apparent discrepancy arises from the composition of each reported dataset. The “MIMIC” and “Hospital” rows in Tables 2–5 represent performance on the entire respective dataset, which necessarily includes the samples used for model training; these full-dataset metrics therefore partly reflect in-sample fit rather than purely independent generalization performance. In contrast, the “MIMIC-test” and “Test set” rows report performance on held-out samples never seen during training, which constitute the genuine measure of the model's generalization ability.

Under single-center validation (Table 2), the MIMIC-test accuracy (0.7550) is lower than the MIMIC-training accuracy (0.8225) and the full MIMIC accuracy (0.8110), consistent with an expected, moderate degree of overfitting typical of deep learning models trained on data of this scale — this is not indicative of a data-processing or modeling error. We have added an explanatory paragraph to the Discussion section clarifying this relationship (see revised manuscript, Discussion, second added paragraph).

We further note that the external Hospital dataset accuracy (0.6925) is lower still than the MIMIC-test accuracy, reflecting a genuine cross-center performance gap rather than an artifact of data splitting; this gap and its plausible explanations are addressed in our response to Comment 2 below.

Comment 2: "Discussion can be elaborated more as to how and why the results obtained are more relevant and of importance and plausible explanations."

Response: We thank the reviewer for this valuable suggestion. We have substantially expanded the Discussion section to provide a more detailed, mechanism-based interpretation of our results, rather than a general summary. Specifically, we now discuss:

(1) Why the cross-center performance gap (MIMIC vs. Hospital) is plausibly attributable to differences in population characteristics, disease severity distribution, and clinical documentation practices between the U.S.-based MIMIC-IV cohort and the Chinese hospital cohort;

(2) Why this gap is asymmetric across modalities: the ablation results show that the textual-modality model's accuracy drops sharply on the Hospital dataset (0.4925, close to chance level) compared with MIMIC (0.6470), while the numerical-modality model degrades much less (0.7240 on MIMIC vs. 0.6875 on Hospital). We attribute this to BioBERT's semantic representations being learned predominantly from English-language biomedical corpora, making the textual modality more sensitive to cross-linguistic and cross-institutional documentation differences, whereas structured numerical variables are inherently more transferable across centers;

(3) Why the multimodal fusion model is more robust than the text-only model in cross-center performance: by combining both modalities, the fused model can rely more heavily on the numerical modality's cross-center robustness while still benefiting from textual semantics when informative, resulting in a proportionally smaller performance loss (MIMIC 0.8110 vs. Hospital 0.6925) than the text-only model alone;

(4) The clinical relevance of these findings: structured numerical indicators appear to be the more transferable and reliable signal for early SA-AKI recognition when a model is to be deployed across institutions or languages, while free-text information adds the most value within a single, consistent documentation system — a consideration directly relevant to future multicenter deployment of the proposed model.

This expanded discussion can be found in the newly added paragraph immediately following the description of the BioBERT-BPNN model's advantages, at the beginning of the Discussion section (see revised manuscript, highlighted text).

We thank the Academic Editor and both reviewers again for their time and valuable feedback, which has substantially strengthened the clarity and depth of our manuscript.

Sincerely,

Yufeng Chen, Hongyang Bian, Yuye Li (Corresponding Author)

Attachment Submitted filename: Response to Reviewers.docx

# Editor report: Decision Letter 1

**DOI:** 10.1371/journal.pone.0357602.r004

**Authors:** Marwan Al-Nimer

19 Aug 2026

Early recognition of sepsis-associated acute kidney injury based on BioBERT-BPNN multimodal clinical data analysis

PONE-D-26-29602R1

Dear Dr. Yuye Li,

We’re pleased to inform you that your manuscript has been judged scientifically suitable for publication and will be formally accepted for publication once it meets all outstanding technical requirements.

Within one week, you’ll receive an e-mail detailing the required amendments. When these have been addressed, you’ll receive a formal acceptance letter and your manuscript will be scheduled for publication.

An invoice will be generated when your article is formally accepted. Please note, if your institution has a publishing partnership with PLOS and your article meets the relevant criteria, all or part of your publication costs will be covered. Please make sure your user information is up-to-date by logging into Editorial Manager at Editorial Manager® and clicking the ‘Update My Information' link at the top of the page. For questions related to billing, please contact billing support.

If your institution or institutions have a press office, please notify them about your upcoming paper to help maximize its impact. If they’ll be preparing press materials, please inform our press team as soon as possible -- no later than 48 hours after receiving the formal acceptance. Your manuscript will remain under strict press embargo until 2 pm Eastern Time on the date of publication. For more information, please contact onepress@plos.org.

Kind regards,

Marwan Salih Al-Nimer, MD, PhD

Academic Editor

PLOS One

Additional Editor Comments (optional):

Reviewers' comments:

# Editor report: Acceptance letter

**DOI:** 10.1371/journal.pone.0357602.r005

**Authors:** Marwan Al-Nimer

PONE-D-26-29602R1

PLOS One

Dear Dr. Li,

I'm pleased to inform you that your manuscript has been deemed suitable for publication in PLOS One. Congratulations! Your manuscript is now being handed over to our production team.

At this stage, our production department will prepare your paper for publication. This includes ensuring the following:

* All references, tables, and figures are properly cited

* All relevant supporting information is included in the manuscript submission,

* There are no issues that prevent the paper from being properly typeset

You will receive further instructions from the production team, including instructions on how to review your proof when it is ready. Please keep in mind that we are working through a large volume of accepted articles, so please give us a few days to review your paper and let you know the next and final steps.

Lastly, if your institution or institutions have a press office, please let them know about your upcoming paper now to help maximize its impact. If they'll be preparing press materials, please inform our press team within the next 48 hours. Your manuscript will remain under strict press embargo until 2 pm Eastern Time on the date of publication. For more information, please contact onepress@plos.org.

You will receive an invoice from PLOS for your publication fee after your manuscript has reached the completed accept phase. If you receive an email requesting payment before acceptance or for any other service, this may be a phishing scheme. Learn how to identify phishing emails and protect your accounts at https://explore.plos.org/phishing.

If we can help with anything else, please email us at customercare@plos.org.

Thank you for submitting your work to PLOS One and supporting open access.

Kind regards,

PLOS One Editorial Office Staff

on behalf of

Professor Marwan Salih Al-Nimer

Academic Editor

PLOS One
